# Transforming Psychiatric Emergency Care: A Community-Focused Model in Trento, Italy

**DOI:** 10.1192/j.eurpsy.2025.605

**Published:** 2025-08-26

**Authors:** W. A. R. Di Napoli, D. Scordato, F. Fasoli, N. Benedetti, C. Agostini

**Affiliations:** 1 Trento Mental Health Center, Azienda Provinciale per i Servizi Sanitari - Trento, Trento, Italy

## Abstract

**Introduction:**

Psychiatric emergencies are a global challenge requiring timely, effective interventions. Traditional intra-hospital approaches often struggle to address the complexity of these crises in a patient-centered and family-inclusive manner. Trento’s Mental Health Service has implemented a community-based, multidisciplinary approach to manage psychiatric emergencies, emphasizing the socio-familial context of each crisis.

**Objectives:**

This study aims to evaluate the effectiveness of the Trento Crisis Team in managing psychiatric emergencies outside of hospital settings, reducing hospital admissions, and enhancing patient and family engagement in the recovery process. Additionally, we assess the impact of the crisis service on public stigma related to mental health crises.

**Methods:**

The study reviews the structure and organization of the Trento Crisis Team, which operates within the Mental Health Centre. The team includes 3 psychiatrists, 5 nurses, 5 educators/psychiatric rehabilitation technicians (TERP), and 5 Peer Support Specialists (*“ESP”* in Italian). Data were collected from emergency intervention records, hospital admission rates, and user satisfaction surveys. Comparisons were made between territorial and intra-hospital crisis management outcomes, with statistical analysis on key performance indicators such as the number of hospital admissions and compulsory health treatments.

**Results:**

Preliminary results indicate a reduction in hospital admissions (*Image 1*) and a significant decrease in the number of compulsory interventions (*Image 2*) since the establishment of the dedicated Crisis Team. While overall user numbers have increased *(Image 3*), the availability of peer support and home-based interventions has improved patient satisfaction and engagement. However, the system still faces challenges in reducing hospital admissions due to the increasing volume of psychiatric emergency cases.

**Image 1:**

**Figure:**
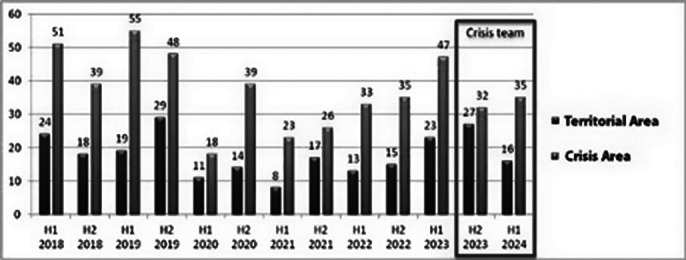

**Image 2:**

**Figure:**
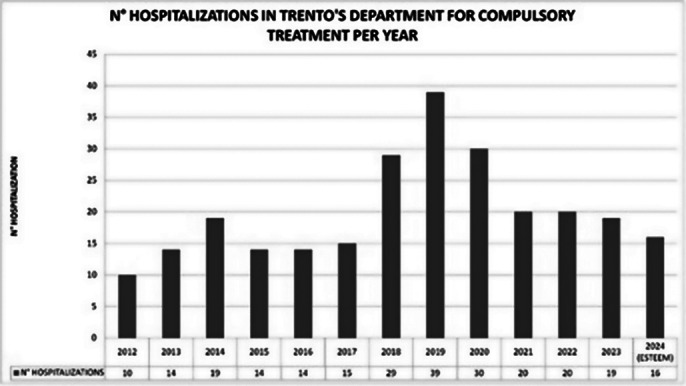

**Image 3:**

**Figure:**
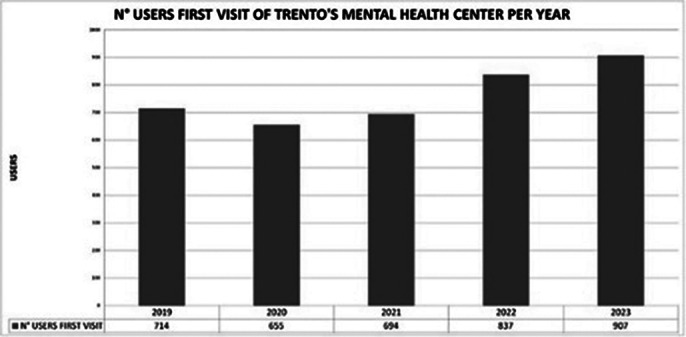

**Conclusions:**

The community-based crisis management model adopted by the Trento Crisis Team offers a promising alternative to traditional hospital-based interventions. By focusing on the individual’s socio-familial environment and engaging Peer Support Specialists, the service has demonstrated a capacity to humanize mental health crises and reduce public stigma. Continued efforts are necessary to address resource constraints and further integrate crisis management into community mental health pathways.

**Disclosure of Interest:**

None Declared

